# Optimizing Nonablative Fractional Laser Treatment: Clinical Evaluation of a Novel 3-Dimentional Controlled Chaos Technology

**DOI:** 10.1093/asjof/ojaf134

**Published:** 2025-10-18

**Authors:** Guy Erlich, Ron Skorochod, Eliran Dahan, Yoram Wolf

## Abstract

The 1550 nm nonablative fractional laser (NAFL) is a well-established and versatile technology in aesthetic dermatology, offering a minimally invasive solution for improvements in skin quality. In this study, the authors introduce a novel NAFL, MOSAIC 3-Dimensional Controlled Chaos Technology (3D CCT), engineered to enhance treatment efficacy and safety. The aim of the authors of this report is to evaluate the safety and efficacy of MOSAIC 3D CCT for facial rejuvenation using patient-reported outcomes, blinded expert assessments, and objective imaging analyses. A retrospective analysis was conducted on 14 patients, aged 34 to 73 years, with Fitzpatrick skin phototypes II to V, who underwent 3 sessions of NAFL treatment for facial rejuvenation at 1- to 2-month intervals. Outcomes were evaluated using the Scientific Assessment Scale of Skin Quality (SASSQ), quantitative analysis with LifeViz imaging software, and the FACE-Q questionnaire. SASSQ analysis showed a trend toward improvement, though none of the changes reached statistical significance (*P* > .05). LifeViz analysis demonstrated improvement in all 5 domains, with statistical significance reached only for wrinkle reduction (*P* ≤ .05). FACE-Q analysis revealed statistically significant improvements across all 12 patient-reported domains (*P* < .05), including clarity, smoothness, radiance, and overall satisfaction. Transient edema and erythema occurred in all patients and resolved within 48 hours. No severe adverse effects, including postinflammatory erythema or hyperpigmentation, were reported. Within the limitations of this small retrospective study, MOSAIC 3D CCT demonstrated potential to improve facial skin quality with a favorable safety profile. Larger prospective studies are needed to confirm these preliminary findings.

**Level of Evidence: 4** (Therapeutic)

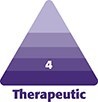

The 1550 nm erbium-glass (Er:Glass) nonablative fractional laser (NAFL) is a well-established technology in aesthetic dermatology.^1-3^ By delivering precisely controlled thermal energy, it generates dermal microthermal zones, triggering a complex wound-healing cascade involving epidermal differentiation, growth factor release, upregulation of collagen and elastin genes, and modulation of inflammation—ultimately driving extracellular matrix (ECM) remodeling and controlled skin regeneration.^4-8^ Histologically, NAFL increases dermal thickness and improves alignment of collagen fibers. Reflectance confocal microscopy findings reveal enhanced dermal papilla density and reduced epidermal irregularities, reflected clinically in reductions in wrinkle depth, smoother skin surfaces, and improved overall skin quality.^9,10^ NAFL promotes rapid re-epithelialization with minimal downtime and a favorable safety profile.^2,11^ The 1550 nm laser is considered safe across all skin phototypes because the wavelength is not absorbed by melanin and is associated with good efficacy and a low incidence of adverse effects, most commonly transient erythema and edema. Although the overall risk of postinflammatory hyperpigmentation (PIH) is low, this risk is elevated in darker skin phototypes, particularly when high-density or high-energy parameters are applied or when cooling is insufficient.^12^

Despite its advantages, NAFL is not without limitations. One key criticism is its relatively modest clinical efficacy compared with ablative fractional lasers (AFLs), often requiring multiple sessions to achieve meaningful improvement.^13,14^ Another significant concern involves adverse effects such as prolonged postinflammatory erythema (PIE) and PIH, particularly when high treatment densities and energy settings are employed, which can result in excessive bulk heating rather than precise thermal injury.^14^

In this study, the authors investigate the efficacy and safety of NAFL for facial rejuvenation using the “MOSAIC 3D Controlled Chaos Technology (3D CCT),” an advanced 1550 nm Er:Glass laser system developed by Cynosure–Lutronic (Seoul, South Korea; Figure 1). Unlike conventional NAFL systems, 3D CCT combines 2 synergistic technological principles. First, the “Chaos” component delivers microbeams in a deliberately nonuniform, randomized pattern to prevent repetitive thermal loading in adjacent tissue. Second, the “3D” component delivers each pulse as a combination of 3 predefined energy levels—high (user-set), medium at 75% of the high setting, and low at 50%—distributed at a fixed density of 200 spots/cm^2^, with respective proportions of 20%, 30%, and 50%. The spatial and depth distribution of these energy tiers is determined by an optimized algorithm. This integration of randomized beam placement with multi-level energy delivery produces controlled, multi-depth dermal coagulation.^15^

**Figure 1.**
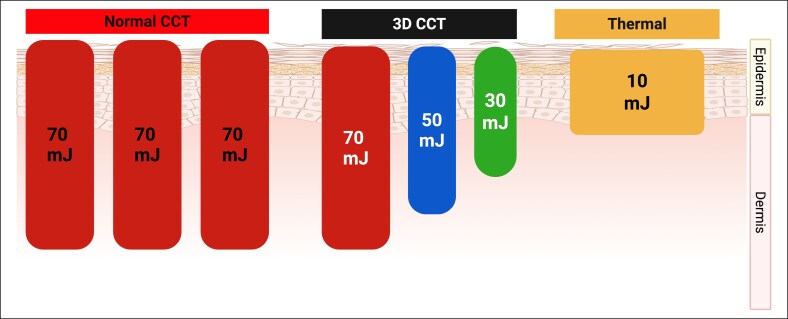
This figure demonstrates the distinct microthermal zone (MTZ) patterns produced by three delivery modes of the novel 1550 nm nonablative fractional laser system, MOSAIC 3-Dimensional Controlled Chaos Technology (3D CCT): standard CCT mode, 3D CCT mode, and thermal mode. In standard CCT mode, uniform high-energy pulses (70 mJ) generate vertically oriented MTZs with consistent depth and width extending into the dermis. The 3D CCT mode, however, delivers a single pulse segmented into 3 energy levels (70, 50, and 30 mJ), resulting in MTZs of variable depths and diameters. In thermal mode, a low-energy pulse (10 mJ) creates a broad, shallow zone of diffuse thermal effect.

## METHODS

### Ethical Considerations

This study was conducted in accordance with the principles outlined in the Declaration of Helsinki and Good Clinical Practice (GCP) guidelines. All patients provided written informed consent prior to treatment, including consent for the use of their anonymized clinical data and images for retrospective analysis, research, and publication purposes. No identifiable information—such as name, identification number, date of birth, or address—was included in the study dataset to ensure complete confidentiality and data protection.

### Patient Selection

In this retrospective study, 14 adult female patients were included, with a median age of 56, ranging from 34 to 73 years, with Fitzpatrick skin phototypes II to V, who sought facial rejuvenation. Inclusion criteria comprised individuals over 18 years of age who had completed a full course of 3 NAFL facial treatments. Patients were excluded if they had undergone any other facial aesthetic procedures during the study period or failed to adhere to the treatment protocol or follow-up schedule.

### Treatment Protocol and Documentation

Each patient underwent 3 treatment sessions, scheduled 29 to 61 days apart. The protocol for each session involved 3 passes with the standard CCT mode, set to Level 3 (50 spots/cm^2^), delivering energy levels of 20, 30, and 40 mJ in the first, second, and third sessions, respectively. This was followed by a single pass of 3D CCT mode (200 spots/cm^2^), delivering energy levels of 30, 40, and 50 mJ, in first, second, and third sessions, respectively, with an average total energy output of 1.425 kJ (0.738-2.387 kJ) per session. All patients applied a topical anesthetic cream 30 min before treatment, composed of Lidocaine 18% and Prilocaine 5% in white paraffin topical ointment (Maayan Haim, Rishon Lezion, Israel), and a cooling device, COOLDECK (Tradis Gat, Israel), was utilized concurrently during the procedure. Immediately after the procedure, a single topical application of BIOGEL (Cellese, AnteAGE, CA) was applied to all patients to support posttreatment healing. All patients were instructed to avoid sun exposure and to use sunscreen (SPF ≥ 50) and emollients throughout the duration of the study. Photographic documentation and FACE-Q questionnaires were obtained at baseline and again 28 to 55 days following the final treatment session, using the LifeViz Mini system (QuantifiCare, France).

### Outcome Measures

To evaluate the impact of NAFL treatment on skin quality, a multimodal assessment approach was employed. Objective evaluation included clinician-based scoring and digital image analysis. First, the Scientific Assessment Scale of Skin Quality (SASSQ) was utilized by 3 blinded, independent experts to assess standardized photographs taken at baseline and after treatment.^16^ Parameters evaluated included elasticity, wrinkles, roughness, pigmentation, erythema, blemishes, and pore size. Evaluators, blinded to time points, assigned scores from 0 (no pathology) to 3 (severe pathology) for each parameter. Second, quantitative analysis was performed using LifeViz imaging software (QuantifiCare, France), which measured changes in wrinkles, skin evenness, pore visibility, oiliness, and pigmentation. Analyses were performed across seven predefined facial regions (forehead, frontal right/left cheeks, lateral right/left cheeks, and lateral periorbital areas) in standardized photographs captured from three angles (frontal, right oblique 45°, and left oblique 45°), using a scoring scale from −10 to +10, where positive values indicate better-than-average skin quality and negative values indicate worse than average. Subjective assessment was obtained through the validated FACE-Q “Satisfaction with Skin” questionnaire, administered at baseline and final follow-up. The questionnaire consisted of 12 items evaluating patient perceptions of facial skin attributes, including: “end of day,” “healthy,” “attractive,” “smooth,” “clear,” “refreshed,” “hydrated,” “wake up,” “radiant,” “tone,” “pores,” and “even-colored.”

### Statistical Analysis

Categorical variables were described as frequencies and percentages out of the whole, whereas continuous variables were described as mean and standard deviation (SD). Chi-sqaured tests or Fisher's exact test were utilized to test categorical hypotheses, and a paired Student's *t* test was utilized for continuous outcomes represented at 2 distinct time points.

## RESULTS

### SASSQ Analysis

Analysis of the outcomes using the SASSQ revealed a trend toward improvement; however, no statistically significant differences were observed across the assessed domains. For elasticity, the mean score increased from 1.93 to 2.02 (*P* = .57). For wrinkles, the mean score decreased from 2.00 to 1.83 (*P* = .25). For roughness, the mean score decreased from 2.17 to 1.88 (*P* = .14). For pigmentation, the mean score increased from 2.52 before to 2.55 after (*P* = .91). For blemishes, the mean score increased from 1.52 to 1.83 (*P* = .12). For pore size, the mean score decreased from 2.05 to 1.93 (*P* = .56). For the overall domain, the mean score decreased from 1.74 to 1.71 (*P* = .89).

### LifeViz Analysis

**Figure 2.**
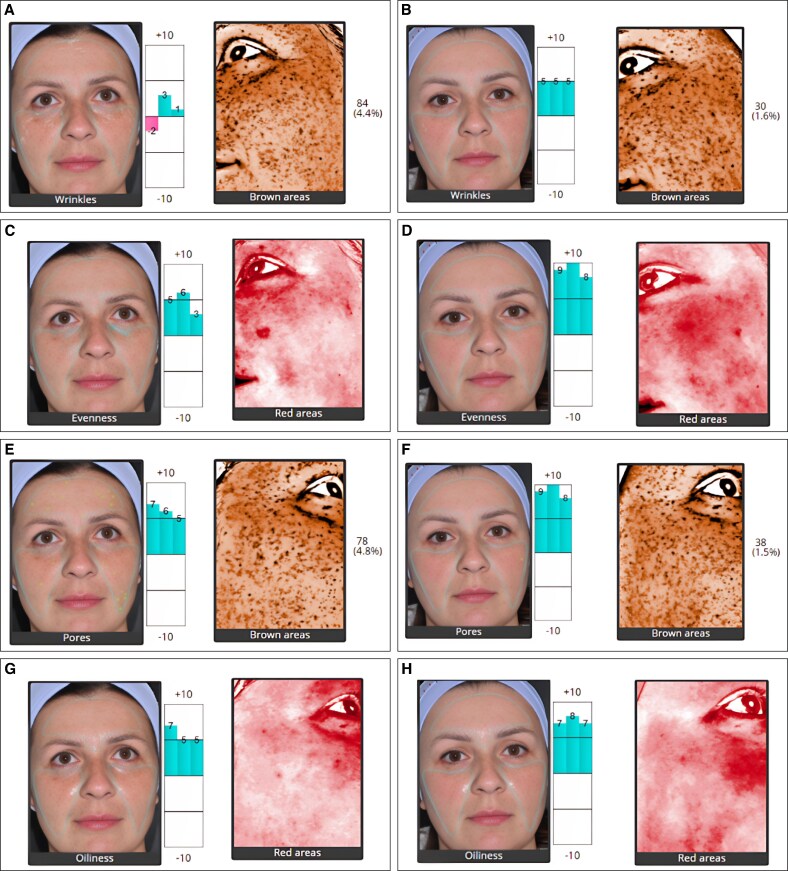
LifeViz analysis of a 34-year-old female patient, showing wrinkles, evenness, pores, oiliness, and brown areas (A, C, E, G) before and (B, D, F, H) after treatment. Comparative assessments are presented, with bar graphs illustrating relative scores that indicate the extent of change.

Across the 5 domains analyzed using the Quantificare software—wrinkles, evenness, pores, oiliness, and brown areas—improvement was noted after treatments when compared with the pretreatment status (Figure 2). Concerning wrinkles, the mean change and corresponding SD differences for the 3 analyzed angles were 3.1 (5.3), 3.2 (5.3), and 3.2 (5.3); for evenness, a mean change of 0.7 (5.4), 0.8 (5.2), and 0.9 (5.2); for pores, 1.1 (5.7), 1.3 (5.5), and 1.4 (5.5); for oiliness, −0.9 (6.3), −0.4 (5.6), and −0.1 (5.2); and for brown areas, −0.44% (1.53%), −0.46% (1.53%), and −0.47% (1.53%). Statistically significant mean differences were noted only in the “wrinkles” domain (*P*-values: .05, .04, and .04).

### FACE-Q Analysis

Analysis of subjective outcomes using the FACE-Q questionnaire demonstrated statistically significant improvements across all 12 domains. For the domain “end of day,” an improvement from a mean score of 2.0 to 3.6 was observed (*P* < .05). For “healthy,” the mean score improved from 2.4 to 3.5 (*P* < .05); for “attractive,” from 2.4 to 3.4 (*P* < .05); for “smooth,” from 2.1 to 3.2 (*P* < .05); for “clear,” from 2.6 to 3.7 (*P* < .05); for “refreshed,” from 2.1 to 3.4 (*P* < .05); for “hydrated,” from 2.3 to 3.2 (*P* < .05); for “wake up,” from 2.1 to 3.1 (*P* < .05); for “radiant,” from 2.0 to 3.1 (*P* < .05); for “tone,” from 2.2 to 3.1 (*P* < .05); for “pores,” from 2.0 to 2.9 (*P* < .05); and for “even-colored,” from 2.1 to 3.0 (*P* < .05).

## DISCUSSION

In this study, the authors assessed the effects of a novel NAFL treatment using a multi-level approach, incorporating patient-reported outcomes, clinician evaluations, and objective digital analysis. FACE-Q questionnaires demonstrated statistically significant improvements across all 12 domains, with patients reporting enhanced clarity, smoothness, radiance, and overall satisfaction. Reviewer-based SASSQ assessments showed a trend toward improvement in elasticity, wrinkles, roughness, and pigmentation, although changes did not reach statistical significance. LifeViz analysis revealed improvements across all 5 parameters, with statistical significance observed only in the wrinkle domain. Although most objective measures did not reach statistical significance, they demonstrated a trend consistent with patient-reported outcomes, supporting the overall direction of clinical improvement. The limited statistical significance in the objective and reviewer-based measurements may be attributed to the relatively small sample size in this study. Larger-scale investigations are warranted to validate these trends and may reveal stronger correlations between subjective satisfaction and quantifiable skin changes.

**Figure 3.**
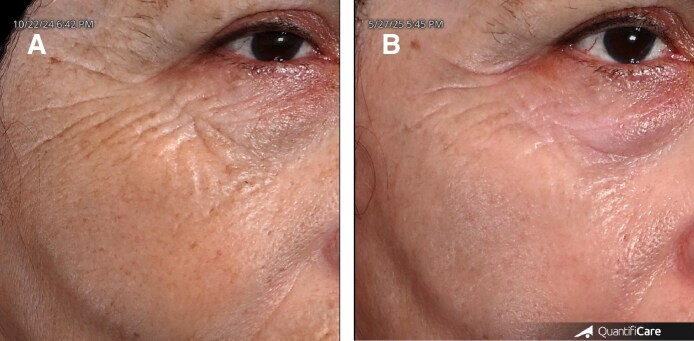
A 63-year-old female patient, shown (A) before treatment and (B) 3 months after completing 3 sessions of MOSAIC 3-Dimensional Controlled Chaos Technology (3D CCT). Treatments were spaced 60 days apart. Each session included 3 passes with the roller handpiece in standard CCT mode (Level 3; 50 spots/cm^2^) at 40 mJ, followed by a single pass with the S20 handpiece in 3D CCT mode at 50 mJ. The average total energy delivered per session was 1.95 kJ. Standardized close-up images of the left periorbital area, captured using the QuantifiCare system, reveal visible improvement in wrinkles, skin texture, and pigmentation.

This retrospective analysis demonstrates the efficacy of MOSAIC 3D CCT laser in improving facial skin quality among patients seeking facial rejuvenation (Figure 3). All patients experienced only transient edema and erythema, which resolved spontaneously within 48 hours. No patient, including darker skin phototype patients, developed any permanent severe side effect, including PIE or PIH, even when higher energy (50 mJ), density (200 spots/cm^2^), and total energy (2.387 kJ) settings were utilized.

The combination of clinical efficacy, minimal downtime, and absence of reported complications supports the potential of the MOSAIC 3D CCT as a notable advancement in what could potentially be dermal remodeling if histologically analyzed. The authors propose that its multilayered energy delivery may enhance the volume of tissue coagulation while reducing its thermal load. This design enables the simultaneous targeting of superficial and deep dermal layers, potentially improving treatment efficacy and promoting more efficient energy distribution, which may help mitigate excessive inflammatory responses.

In this small cohort, no clear association was identified between patient age, skin phototype, or baseline severity of skin laxity and the extent of clinical improvement. Given the limited sample size, these findings should be interpreted with caution, and future larger-scale studies are needed to explore these potential associations more robustly.

Despite these promising findings, this study has limitations. The relatively small sample size and lack of long-term follow-up limit the generalizability of the results. Future studies with larger cohorts and extended follow-up periods, incorporating histological analysis, would provide a more comprehensive understanding of the laser's regenerative mechanisms.

The 1550 nm NAFL MOSAIC 3D CCT demonstrated both efficacy and safety in enhancing skin quality, as evidenced by objective assessments and patient-reported outcomes. These findings highlight its value as a key modality in aesthetic dermatology, offering an efficient, minimally invasive approach to facial rejuvenation with minimal downtime and a favorable safety profile.

## CONCLUSIONS

This early clinical experience with MOSAIC 3D CCT suggests that the device may improve facial skin quality, as supported by patient-reported outcomes, blinded expert evaluations, and objective imaging analyses. Treatments were generally well tolerated, with only transient erythema and edema observed, and no PIH or erythema reported. Although these preliminary results indicate a favorable safety profile and potential clinical value, the small sample size limits definitive conclusions. Larger prospective studies are needed to confirm these findings and further establish the role of this technology in nonablative fractional skin rejuvenation.
